# Supplementary material for: Surface Mobility of a Glass-Forming Polymer in an Ionic Liquid
Source: Macromolecules. 2025 Dec 4;58(24):13119–24. doi: 10.1021/acs.macromol.5c01895 (PMC12752691; doi:10.1021/acs.macromol.5c01895)
Supplement: Supplementary file 1 [file ma5c01895_si_001.pdf]

Supporting Information - Surface mobility of a glass-forming polymer in an ionic liquid

Xinyu Zhang,[1] Christian Pedersen,[3,4] Haoqi Zhu,[1] Siming Wang,[1] Yuchen Fu,[1] Liang Dai,[1] Andreas Carlson,[3,5] Thomas Salez,[2,*] and Yu Chai [1,*]

[1] *Department of Physics, City University of Hong Kong, 83 Tat Chee Avenue, Kowloon, Hong Kong SAR, China.*
[2] *Univ. Bordeaux, CNRS, LOMA, UMR 5798, Talence F-33400, France.*
[3] *Mechanics Division, Department of Mathematics, University of Oslo, 0316 Oslo, Norway.*
[4] *Expert Analytics AS, N-0179 Oslo, Norway.*
[5] *Department of Medical Biochemistry and Biophysics, Umeå University, Umeå, Sweden.*
[*]Corresponding authors: yuchai@cityu.edu.hk; thomas.salez@cnrs.fr

SECTION 1. Long-Range Interactions Between the Ionic Liquid and PS Films

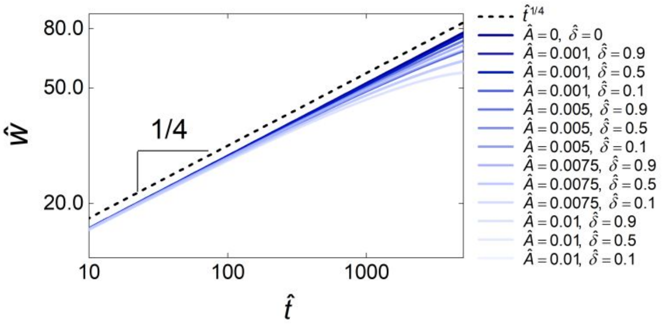

Fig. S1. The non-dimensional width $\hat{w}$ as a function of non-dimensional time $\hat{t}$. The data follows the expected $\hat{t}^{1/4}$ power-law, which is plotted as the black dashed line, for a long time before deviating. The maximum time measured in the experiments is $\hat{t}$< 100.

Fig. S1 shows the evolution of the non-dimensional step width $\hat{w}$ as a function of non-dimensional time $\hat{t}$. Dimensionless variables

$$\hat{A} = A/\left(6\pi\gamma_{\text{PS-l}}\, h_0{}^2\right), \tag{1}$$

$$\hat{\delta} = \delta/h_0\,, \tag{2}$$

$$\hat{t} = t\gamma_{\text{PS-l}}\, h_{\text{m}}{}^3/(3\eta h_0{}^4\,), \tag{3}$$

$$\hat{h} = h/h_0\,, \tag{4}$$

where h_0 is the initial thickness of the bottom PS layer, here we fix h_0 = 78 nm. The results show that it takes a long time, when comparing to the maximum experimental measurement time which is $\hat{t}$<100, and large values of $\hat{A}$ for $\hat{w}$ to deviate from the expected $\hat{t}^{1/4}$.power. So even though this can lead to a slightly better fit it does only modify the mobility slightly. At most we observed a ~10% change in mobility for a given experiment which can rule out the existence of such strong long-range interactions between the ionic liquid and PS films.

SECTION 2. Capillary Thin-Film Equation with Different Free Surface Boundary Conditions

Considering the dynamics of a thin viscous film of thickness $h(x,t)$ and length L, in which the vertical length scale is much less than the horizontal, we get a thin film Reynold's number $\text{Re}_{tf} = \text{Re}_{h/L} \ll 1$. Thus, fluid inertia can be neglected, and we can apply the lubrication theory which yields the leading order momentum equations:

$$\frac{\partial p}{\partial z} = 0 \tag{5}$$

$$\frac{\partial p}{\partial x} = \eta \frac{\partial^2}{\partial z^2} u \tag{6}$$

with $p(x,t)$ being the pressure, η is the films viscosity, u (x,t) is the horizontal flow velocity and x, z are the horizontal and vertical coordinates, respectively. As the film is resting on a solid substrate we impose a no-slip condition at the film-substrate interface. However, at the film's free interface it is not obvious what condition to apply. Depending on the ambient conditions our options are a no-slip condition or a no-shear condition. The two alternatives yield two different solutions to the velocity field:

$$u_{\text{no-slip}} = \frac{1}{2\eta}\frac{\partial p}{\partial x}(z^2 - h^2) \tag{7}$$

$$u_{\text{no-shear}} = \frac{1}{2\eta}\frac{\partial p}{\partial x}(z^2 - 2zh) \tag{8}$$

We can now integrate the continuity equation by applying the Leibniz integral rule to obtain

$$\frac{\partial h}{\partial t} = -\frac{\partial\left(\int_0^h u\,dz\right)}{\partial x} \quad (9)$$

For the flow fields originating from our two options of boundary conditions, this yields the thin film equations

No-slip:

$$\frac{\partial h}{\partial t} = \frac{1}{12\eta}\frac{\partial}{\partial x}\left(h^3\frac{\partial p}{\partial x}\right) = 0 \quad (10)$$

No-shear:

$$\frac{\partial h}{\partial t} = \frac{1}{3\eta}\frac{\partial}{\partial x}\left(h^3\frac{\partial p}{\partial t}\right) = 0 \quad (11)$$

Similar, yet different by a pre-factor.

SECTION 3. Determination of the Width of Stepped Films

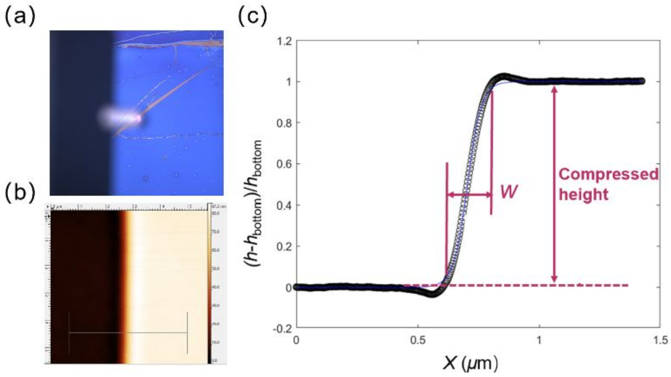

Fig. S3. (a) Optical microscope image of 50 nm +50 nm PS film, the blue region is the bottom layer film only, the yellow area represents the bottom layer coated with the top layer film. AFM scans the boundary between the two regions to obtain a stepped profile. (b) AFM morphology image of the stepped film. (c) The film profile is read by the MATLAB program and fitted with a *tanh*[x/(w/2)] function to obtain the value of the so-called width w.

SECTION 4. Determination of the T_g of Stepped Films

The molecular weight dependent T_g provided by Polymer Source Inc. (https://www.polymersource.ca/index.php?route=product/category&path=2_2183_14_90_2189_503&product_id=6705&subtract=1&serachproduct=yes&categorystart=A-1.1)
